# Antidiabetic Effects of a Short Peptide of Potato Protein Hydrolysate in STZ-Induced Diabetic Mice

**DOI:** 10.3390/nu11040779

**Published:** 2019-04-04

**Authors:** Shibu Marthandam Asokan, Ting Wang, Wei-Ting Su, Wan-Teng Lin

**Affiliations:** 1 Department for Management of Science and Technology Development, Ton Duc Thang University, Ho Chi Minh City 700000, Vietnam; marthandam.asokan.shibu@tdtu.edu.vn; 2 Faculty of Applied Sciences, Ton Duc Thang University, Ho Chi Minh City 700000, Vietnam; 3 Department of Hospitality Management, College of Agriculture, Tunghai University, Taichung 40704, Taiwan; s870106@yahoo.com.tw; 4 Department of Food Science, College of Agriculture, Tunghai University, Taichung 40704, Taiwan; rd2@hansient.com.tw

**Keywords:** diabetes mellitus, alcalase, APPH, bioactive peptides, hyperglycemia, β-cell

## Abstract

Alcalase- generated potato protein hydrolysate (APPH) is a potential bioactive peptide against diabetes mellitus (DM) and DM-associated secondary effects in animal models. The aim of the present study was to find the efficiency of a deca-peptide DIKTNKPVIF (DF) from APPH against DM. Six-week-old male ICR mice were divided into the following groups: Control, Control+DF (received 50 mg/kg DF), streptozotocin (STZ)-induced DM group, DM+Acarbose group (20 mg/kg of acarbose), DM+DF-L (25 mg/kg of DF), DM+DF-H (50 mg/kg of DF), and DM+APPH (50 mg/kg of APPH). Comparable to APPH, treatment with DF effectively regulated blood glucose level and also controlled plasma total glycerol (TG), total cholesterol (TC), insulin, and HbA1c levels in DM animals. DF treatment also showed evidence of ameliorating DM-associated damages in the pancreatic islets and in the liver, heart, and kidney tissues. Therefore, the results demonstrate that the short synthetic peptide-DF may effectively provide protection against DM-associated damages.

## 1. Introduction

Diabetes mellitus (DM) is a widespread disease that affects 30.3 million people in the United States and is estimated to cause 1.5 million deaths around the world [1,2]. There has been a rapid increase in the global diabetic population, which is projected to reach epidemic proportions in many developing countries. Type one DM (T1DM or insulin-dependent diabetes mellitus) is a chronic and progressive disease that results from damaged pancreatic β-cells that secrete insulin [3,4]. Supplementation of insulin is the only effective clinical management strategy available for T1DM. 

T1DM is characterized by elevated fasting blood glucose levels, which is also associated with defects in β-cell mass and results in damaging the structure and function of various organs. The pathological process of the selective destruction of pancreatic β-cells, referred to as insulitis, is mediated by inflammatory events in the islets of Langerhans. Streptozotocin (STZ) is commonly used to induce T1DM rodent models that mechanistically resemble insulitis [5,6]. A single dose of high-dose STZ induces T1DM as a result of β-cell depletion [7].

Hyperglycemic condition causes disturbances in the biochemical and cellular homeostasis that are hallmarks of DM [8,9]. Such imbalances induced by hyperglycemia also cause profound apoptosis of β-cells and inflammation-associated insulitis, resulting in the depletion of β-cell mass and their function [10]. Deterioration of β-cell mass subsequent to hyperglycemia is significant, as clinical implications of T1DM are apparently observed only when ~80% of the β-cells are destroyed [11]. As there are not many reliable strategies to overcome β-cell damages, considerable effort has been made to develop novel therapeutic interventions in order to preserve their population and function. While anti-inflammatory agents like curcumin show positive effects, with poor bioavailability their applications are limited. Further, the pathological implications of T1DM also include microvascular complications such as nephropathy, neuropathy, and retinopathy, and macrovascular complications such as coronary heart disease [4]. Patients with T1DM are at high risk for cardiovascular diseases; according to recent predictions, about 17% of T1DM patients experience cardiovascular events within 10 years of disease diagnosis [12]. Therefore, management of such complications is an integral part of the management strategies for T1DM. 

Evidence shows that bioactive proteins and peptides from various sources possess various physiological functions, and their possible therapeutic applications have been explored extensively [13,14,15,16,17,18]. Gastrointestinal digestion of proteins to simpler forms of peptide results in molecules with similar bioactivities as their parent proteins [19,20]. Enzymatic hydrolysis of proteins generates smaller peptides and free amino acids and therefore improves the nutritional value, safety, and function of the food [21,22]. These hydrolysates contain small peptides that potentially regulate vital functions in the body through various biological properties such as antihypertensive, antithrombotic, antioxidant, and immunomodulatory activities [22,23]. The functions of the bioactive peptides are carried by specific amino acid sequences that interact with other proteins to alter their function [22]. While the structure–function relationships of bioactive peptides are not clearly known, they are generally 2 to 20 amino acids long and contain mostly hydrophobic amino acids [22].

Potato (*Solanum tuberosum* L.) is a major source of dietary proteins, minerals, and antioxidants [24,25]. An alcalase generated potato protein hydrolysate (APPH) derived with lipolysis-stimulating activity was found to have the potential to act as an efficient anti-obesity diet ingredient; APPH was found to be effective at reducing diabetic damages to the heart and liver by attenuating cell apoptosis and activating mitochondrial biogenesis by restoring SIRT1 expression that was curtailed by high-fat diet [26]. In further studies we confirmed that a peptide fragment DIKTNKPVIF (DF) was consistently present in the MS analysis of APPH [26]. Therefore, in this study we focused on the effect of DF on the effects of diabetes in STZ-induced DM mice. The results show that the peptide treatment is effective in preserving the β-cell mass by regulating inflammatory mediators and thereby regulating hyperglycemia in STZ-induced DM mice.

## 2. Material and Methods

### 2.1. Chemicals

All the chemicals were purchased from Sigma-Aldrich (St. Louis, MO, USA) unless specified otherwise. APPH was prepared as mentioned in our previous reports [26]. DF peptide was commercially synthesized by DGpeptides Co.,Ltd. (Zhejiang, China). ELISA kits for glycosylated hemoglobin (HbA1_c_) and insulin were from Beijing BHKT Clinical Reagent Ltd, (Beijing, China) and those for Interleukin (IL)-1β and IL-10 were purchased from R&D systems (Minneapolis, MN, USA). Antibodies were purchased from Santa Cruz Biotechnology (Dallas, TX, USA). 

### 2.2. Animal Experiments

This study was conducted following the IACUC-10518 protocol and approved by the institutional animal care and use committee (IACUC) of National Taiwan Sport University, Taiwan. One week prior to the experiments, the mice (six-week-old male ICR) were allowed to adapt to the environment and the diet. During the one-week adaptation period, all the animals were housed in a room maintained at 24 ± 2 °C and 55 ± 10% humidity with a 12 h light cycle. The mice were randomly assigned into seven groups (*n* = 8): non-diabetic normal mice (Control); a non-diabetic mice treated with 50 mg/kg of DIKTNKPVIF (Control+DF); STZ-induced diabetic control (DM); DM mice with acarbose (20 mg/kg/day) treatment (DM+Acarbose); DM mice with low-dose (25 mg/kg/day) DIKTNKPVIF peptide treatment (DF-L); DM mice with high-dose (50 mg/kg) DIKTNKPVIF treatment (DF-H); and DM mice with APPH (50 mg/kg/day) treatment (DM+APPH). The animals were fed with a standard laboratory diet (PMI Nutrition International, Brentwood, MO, USA) and were provided with reverse osmosis-treated water ad libitum. The peptides were diluted in saline by equalizing the protein content in defined volume of aliquots with Bovine serum albumin (BSA). After adaptation period, one shot of STZ (60/mg/kg, IP) was administered and from the following day the peptide treatment was given every day for 4 weeks using oral gavage; blood samples for oral glucose tolerance test (OGTT) were collected from tail vein after two weeks and at the end of the four weeks treatment schedule. The animals were then euthanized by terminal anesthesia and decapitation; further, the spurted blood was collected for biochemical analysis. 

### 2.3. Hemotoxylin and Eosin Staining

The tissue sections were prepared from excised organs that were soaked in formalin. The tissue sections were dehydrated by passing them consecutively through graded alcohol and were then embedded in paraffin wax. The embedded tissue blocks were then cut into 0.2 μm-thick sections and de-paraffinized by soaking in xylene. For adipose tissue, adipose tissues that were removed from the dorsal wall of the abdomen were fixed in 10% formaldehyde/ Phosphate Buffered Saline (PBS) and dehydrated, embedded in tissue-freezing medium (Tissue-Tek OCT compound; Miles Inc., Elkhart, IN, USA), and frozen in dry ice and acetone. The adipose tissue was cut into 10-μm sections [4]. All the tissue slices were stained by hematoxylin and eosin (H&E) and rinsed with water. Photomicrographs were obtained using Zeiss Axiophot microscopes (Carl Zeiss Microscopy, Thornwood, NY, USA). The cell area was determined by using ImageJ image processing program (National Institutes of Health, Bethesda, MA, USA). 

### 2.4. Determination of Biochemical Profile

Blood samples were collected from the test mice and placed in centrifuge tubes containing heparin (10 μL, 1000 IU mL−1). The blood plasma was separated by centrifugation at 10,000 rpm for 10 min; triglyceride (TG), Low-density lipoprotein cholesterol (LDL-C), total cholesterol (TC), IL-1β, and IL-10 levels were measured by using commercially available assay kits.

### 2.5. Immunohistochemistry

For immunohistochemical staining, the slides were kept in boiling citrate buffer (pH 6.0) for 30 min and were then blocked with serum and then incubated with primary antibody overnight at 4 °C. Biotinylated secondary antibody was applied on the sections and left for 1 h at room temperature. The sections were treated with DAB Substrate (Hoffmann-La Roche Ltd, Basel, Switzerland) for 5 to 15 min, and photographs were taken using an Olympus P74 (Olympus, Waltham, MA, USA) microscope for analysis. 

### 2.6. Statistical Analysis

Quantitative data are shown as the mean ± SD corresponding to three or more replicates. Analysis of statistical differences was done by one-way ANOVA with Tukey’s post hoc analysis using GraphPad Prism 4 software (GraphPad Software Inc., San Diego, CA, USA). A *p*-value of < 0.05 was considered statistically significant.

## 3. Results

### 3.1. Effect of STZ-Induced DM on Diet Uptake

**Figure 1:**
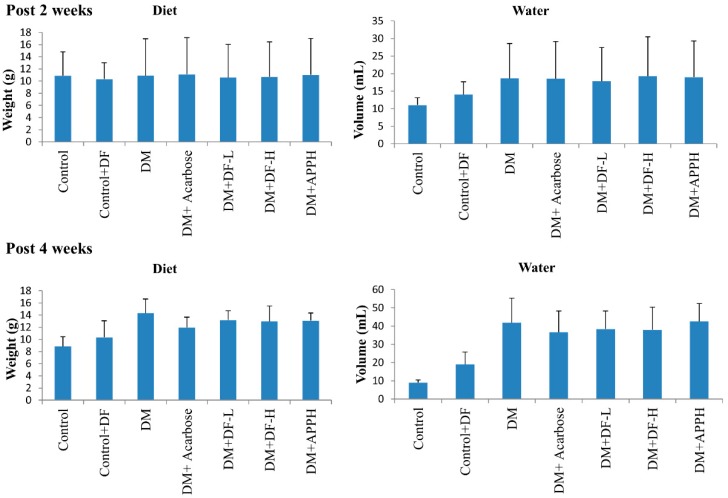
Effect of DIKTNKPVIF (DF) on diet intake. The changes in feed and water intake in non-diabetic control mice (Control), non-diabetic mice treated with 50 mg/kg of DF (Control+DF), STZ-induced diabetes mellitus (DM) mice, DM mice treated with acarbose (DM+Acarbose), DM mice treated with low-dose DF peptide (DM+DF-L), DM mice treated with high-dose DF peptide (DM+DF-H), and DM mice treated with alcalase generated potato protein hydrolysate (APPH) peptide (DM+APPH) after 2 weeks and after 4 weeks of STZ injection. The results represent mean ± SD from 8 mice.

The feed and water uptake of mice in the first two weeks after STZ injection did not show any significant difference with respect to the control groups. However, after 2 weeks the STZ-induced mice consumed slightly higher levels of feed and water, which was not affected by treatment with the peptides (Figure 1). Control groups treated with DF did not show any significant changes in diet uptake. 

### 3.2. Effect of DF on Glucose Tolerance

**Figure 2:**
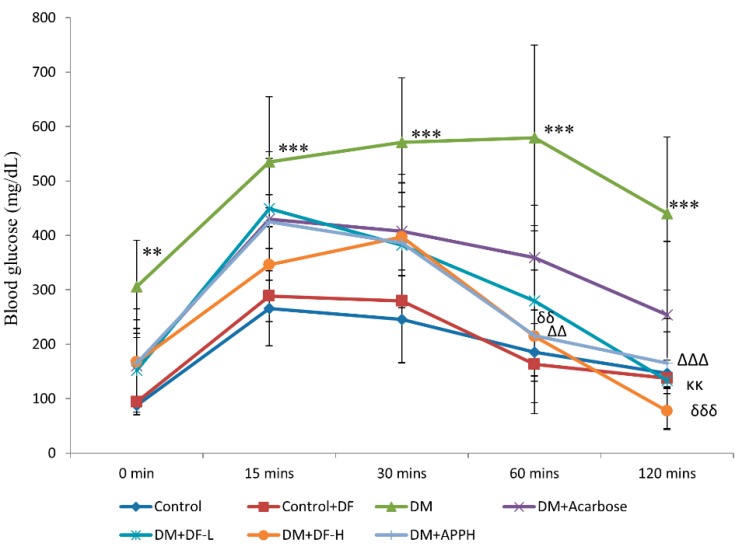
Changes in blood glucose level observed with oral glucose tolerance test (OGTT). OGTT results after 4 weeks of treatment show differences in blood glucose levels in non-diabetic controls (Control), non-diabetic mice treated with DF (Control+DF), STZ-induced diabetes mellitus mice (DM), DM mice treated with acarbose (DM+Acarbose), DM mice treated with low-dose DF peptide (DM+DF-L), DM mice treated with high-dose DF peptide (DM+DF-H), and DM mice treated with APPH peptide (DM+APPH) after 15 min, 30 min, 60 min, and 120 min of oral glucose administration. ** = *p* < 0.01 and *** = *p* < 0.001 indicate significant difference in DM group with respect to the control group; κκ = *p* < 0.01 indicates significant difference in DM+DF-L group with respect to the DM group. δδ = *p* < 0.01 and δδδ = *p* < 0.001 indicate significant difference in DM+DF-H group with respect to the DM group. ∆∆ = *p* < 0.01 and ∆∆∆ = *p* < 0.001 indicate significant difference with respect to the DM group.

The OGTT results performed after 4 weeks of treatment show that STZ-induced DM mice showed significant elevation and stability of blood glucose levels with respect to time. However, in the STZ-injected mice administered with the DF peptide, a notable reduction in the glucose level was observed that was comparable to the potato protein hydrolysate APPH, which was shown to be an effective control of blood glucose (Figure 2).

### 3.3. DF on Serum Parameters

**Figure 3:**
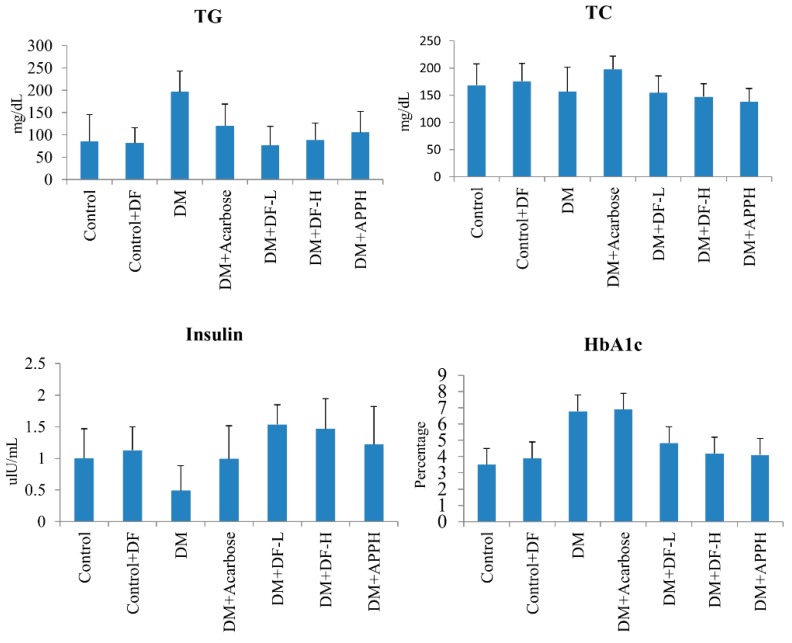
Effects of DF peptide on biochemical changes in the blood plasma. The results of a biochemical analysis show the difference in plasma triglycerides (TG), total cholesterol (TC), insulin, and HbA1c in non-diabetic controls (Control), non-diabetic mice treated with DF (Control+DF), STZ-induced diabetes mellitus mice (DM), DM mice treated with acarbose (DM+Acarbose), DM mice treated with low-dose DF peptide (DM+DF-L), DM mice treated with high-dose DF peptide (DM+DF-H), and DM mice treated with APPH peptide (DM+APPH).

Parameters of lipid metabolism in the blood, such as total glycerol (TG) and total cholesterol (TC), and diabetes-associated parameters, such as the levels of insulin and HbA1c, were found to be modulated in the DM animals, thereby showing the disruption of the metabolic homeostasis in DM mice. However, treatment with DF regulated the levels of serum TG and TC in a dose-dependent manner. Further, the level of insulin and HbA1c was also found to be conserved in the animals treated with DF or with APPH (Figure 3).

### 3.4. DF on Diabetes-Associated Pathological Events

**Figure 4:**
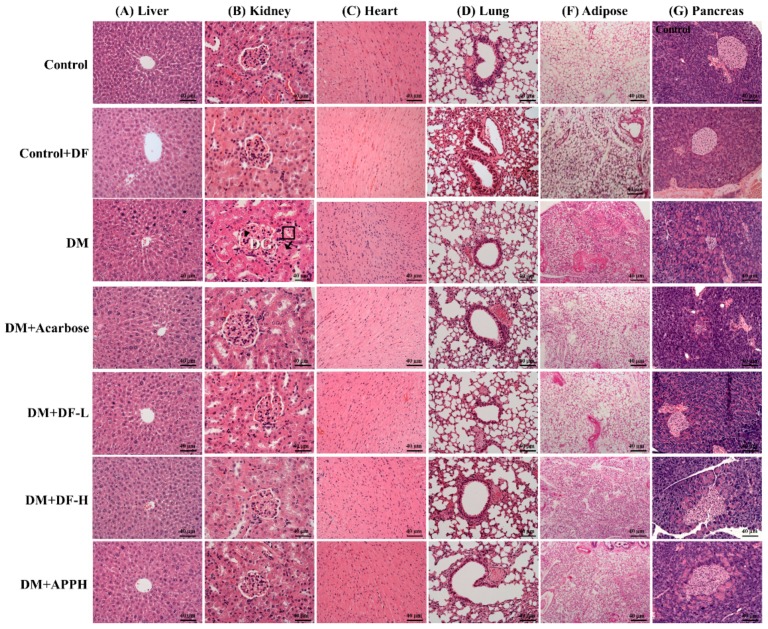
DF-induced changes in the histopathology of DM animals. Hematoxylin and eosin (H and E) stainings of tissue sections from liver, kidney, heart, lung, adipose tissue, and pancreas show differences in the histopathology in control mice (Control), non-diabetic mice treated with DF (Control+DF), STZ-induced diabetes mellitus mice (DM), DM mice treated with acarbose (DM+Acarbose), DM mice treated with low-dose DF peptide (DM+DF-L), DM mice treated with high-dose DF peptide (DM+DF-H), and DM mice treated with APPH peptide (DM+APPH).

The DM mice treated with DF and APPH showed a higher protection against diabetes-induced pathological changes in the organs such as the heart, kidney, and pancreas (Figure 4). Investigation of the liver tissue sections showed normal tissue architecture in the control group animals, but the DM groups showed loss of normal hepatocyte architecture, necrosis, apoptotic nuclei, hydropic degeneration, and congestion of the central vein. However, the treatment groups showed significant improvement in the liver morphology, showing amelioration of the DM effects in the liver (Figure 4A). Kidneys of DM mice showed accelerated renal mesangial expansion (arrowheads in Figure 4B), an important characteristic of diabetic nephropathy. Morphological changes in the DM kidneys also included hypertrophied and distorted glomeruli (DG in Figure 4B) and tubular necrosis (shown in squared box in Figure 4B), and vacuolization of the glomerular matrix, all of which were absent in the control mice. After 4 weeks of treatment with DF and APPH, the pathological morphology changes were ameliorated effectively (Figure 4). 

The heart tissue section showed a notable hypertrophic effect in the cardiomyocytes with disorientation in the tissue arrangement and increased interstitial spaces. However, in the animal groups treated with DF the pathological changes were limited and were comparable to the APPH treatment group, which was also better than the acarbose treatment group. The lung tissue section showed that while the control group had normal alveolar and bronchioles, the DM groups showed infiltration of lymphocytes. The size of adipocytes in the subcutaneous adipose tissue in the abdomen was also found to be reduced in the STZ-induced diabetic animals. However, treatment with DF effectively reduced the effects of STZ-induced diabetes. Further, the pancreatic tissue section showed that the pancreatic islets of the normal mice appeared as rounded areas with cells arranged in irregular, branching, and anastomosing cords separated by blood capillaries, whereas in the diabetic group the pancreatic islets showed degenerative changes, particularly in the islets, with a reduction in their size and number.

### 3.5. Immunohistochemical Evaluation

**Figure 5:**
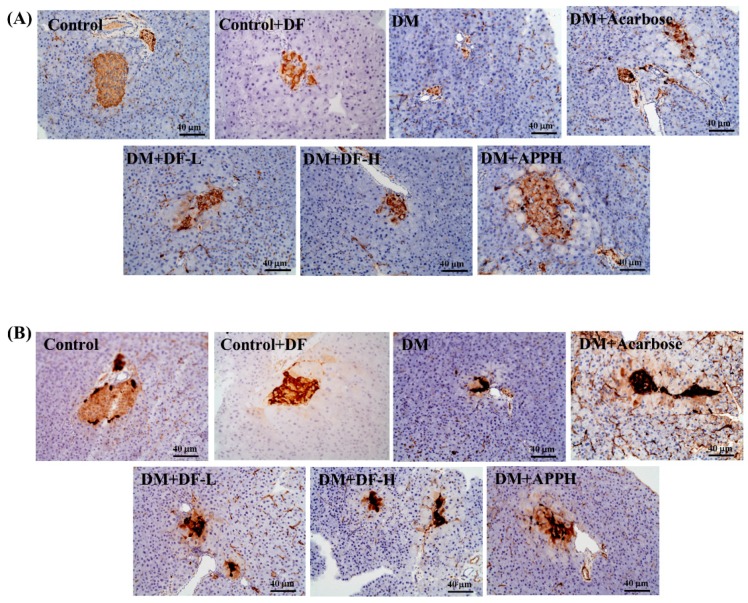
Changes in the DM-induced modulations in glucagon and insulin secretion. Immunohistochemical staining for insulin (**A**) and glucagon (**B**) in control mice (Control), non-diabetic mice treated with DF (Control+DF), STZ-induced diabetes mellitus mice (DM), DM mice treated with acarbose (DM+Acarbose), DM mice treated with low-dose DF peptide (DM+DF-L), DM mice treated with high-dose DF peptide (DM+DF-H), and DM mice treated with APPH peptide (DM+APPH).

In the control groups, the insulin-secreting β-cells and those cells expressing glucagon formed the major population of cells in the islets. Positive insulin secretion was observed as dark brown in the cytoplasm of β-cells. In the DM groups, there was marked reduction of β-cells expressing insulin. However, in the groups treated with DF or with APPH there was a clear increase in the population of cells expressing insulin (Figure 5). 

### 3.6. Anti-Inflammatory Effects of DF in STZ-Diabetic Mice

**Figure 6:**
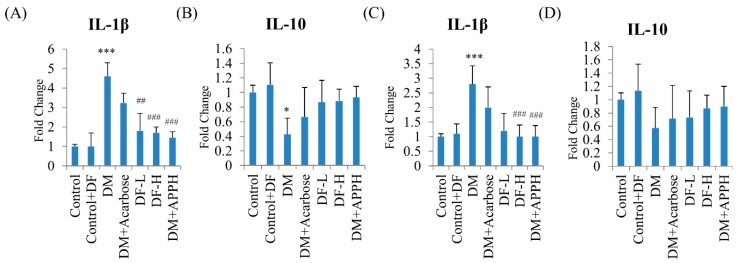
DF suppressed modulated inflammatory cytokines in DM mice. ELISA assay of the pancreatic (**A**,**B**) and the serum (**C**,**D**) levels of IL-1β (**A**,**C**) and IL-10 in non-diabetic controls (Control), non-diabetic mice treated with DF (Control+DF), STZ-induced diabetes mellitus mice (DM), DM mice treated with acarbose (DM+Acarbose), DM mice treated with low-dose DF peptide (DM+DF-L), DM mice treated with high-dose DF peptide (DM+DF-H), and DM mice treated with APPH peptide (DM+APPH). * = *p* < 0.05 and *** = *p* < 0.001 indicate significant difference with respect to the control group; ## = *p* < 0.01 and ### = *p* < 0.001 indicate significant difference with respect to the DM group mice.

To further identify the mechanism underlying the antidiabetic effects of DF, the serum and pancreatic levels of the inflammatory cytokines IL-1β, which exaggerates β-cell apoptosis, and IL-10, which averts insulitis, were analyzed. ELISA assays revealed a significant elevation of IL-1β in the pancreas and in the serum of STZ-induced diabetic mice (Figure 6). However, treatment with different concentrations of DF effectively regulated the levels of IL-1β similar to that observed in the groups treated with APPH. Meanwhile, the pancreatic and serum IL-10 levels, which were low in the DM mice, were found to be normal in the treatment group, similar to that of the control group (Figure 6). 

## 4. Discussion

APPH is a lipolytic peptide with efficient cardio-protective and hepato-protective effects and with proven anti-aging potential [26,27,28]. It is known to attenuate lipid accumulation, apoptosis, and fibrosis in liver and reduces serum triglycerides, total cholesterol, and low-density lipoprotein levels, and thereby inhibits extrinsic apoptosis in the hearts of aging mice [26,27,28].

Potato proteins are associated with abundant nutritional value and a balanced hydrophilic/hydrophobic amino acid composition [29,30,31]. Usually the methods of recovering proteins from potato include high temperature treatments that cause reductions in yield and functional properties of the potato proteins [32]. Therefore, converting the insoluble protein into functionally active, value-added ingredients would enrich it as a functional food. Enzymatic hydrolysis of potato proteins is a possible alternative strategy to produce functionally viable potato protein and peptides [33]. Partial enzymatic cleavage of the proteins under proper conditions potentially produces soluble proteins with potential bioactivity. The bioactivity of APPH from potato proteins is attributed to the diversity of the generated peptide fragments exposing reactive amino acid side chains or hydrophobic patches [34]. DF is an abundant peptide fragment found in APPH with two possible aliphatic side chains with the presence of isoleucine [18,26].

Our results show that APPH and DF treatment exerts an antidiabetic effect in STZ-induced diabetic mice. STZ is normally used to induce T1DM, causing toxicity to β-cells of the pancreatic islets. As in human DM, STZ-induced DM also causes elevated diet and water uptake, and regulation in blood glucose is known to reverse the trend. However, our results show that regulation of DM by APPH and DF did not show any correlation with water uptake, but a slight increase in water intake in the control group treated with DF reflects that there may be a difference in the palate towards peptide solution and plain saline, causing excessive water uptake in the treatment groups. 

Hyperglycemic condition in DM patients is known to trigger oxidative stress and cytotoxicity in the pancreatic cells, causing dysfunction of insulin-secreting β-cells. STZ-induced DM animals exhibit similar pathophysiological phenomena as seen in diabetic humans. Conditions such as hypoinsulinemia as well as liver and cardio-renal dysfunctions are the usual impacts of diabetes [4].

The role of IL-1β in inducing β-cell dysfunction by triggering apoptosis is well known in diabetic conditions [35]. The role of IL-1β is evident in both types of DM and is known to activate nuclear factor-κB (NF-κB)-associated inducible nitric oxide synthase (iNOS) to generate nitric oxide (NO), which mediates inhibition of insulin secretion in pancreatic islets [36]. On the contrary, IL-10 contributes to the reduction of insulitis in pancreatic tissues [37]. Our results suggest that APPH and DF possess efficient immunomodulatory effects to bring IL-1β and IL-10 to normal levels, which may contribute to the functional restoration of pancreatic β-cells. It is also notable that APPH displays anti-apoptotic effects in heart and liver tissues of high-fat diet-induced obesity models [26,27]. In correlation with previous reports, APPH and its constituent DF show protective effects against the DM-induced deterioration of pancreatic cells. 

Normal histopathological appearance with organized islets and elevated insulin-expressing cells in the islet structures of the DF treatment group when compared to the DM mice show that DF is an important constituent of APPH with anti-diabetic potential. As expected, the results show that APPH is highly efficient in maintaining the insulin-secreting β-cell population. Pancreatic cells are formed by neogenesis during gestation and by replication of differentiated pancreatic stem cells after birth [38,39,40]. β-cell mass is one of the determining factors of the total insulin secretion and therefore maintenance of at least a partial β-cell population is essential to revert T1DM conditions [41,42]. Administration of APPH and its constituent DF effectively preserved the β-cell population that survived the STZ assault and further facilitated the revival of the β-cell mass. The regulation of blood glucose level in the treatment groups is also possibly due to the conserved β-cell population that produces insulin. 

Fasting blood glucose and HbA1c levels are crucial parameters to diagnose diabetes patients who are more prone to coronary heart diseases, ischemic stroke, and hepatic and renal diseases. HbAlc levels are known to possess a direct correlation with the blood glucose levels [43]. In this study, the levels of HbA1c that were elevated in the DM groups were significantly reduced following treatment with DF, similar to the effect displayed by APPH. The effect on HbA1c may be potentially due to the restoration of insulin secretion by the conserved β-cell population. STZ induced elevation in the plasma TG and TC, which were regulated when administered with DF and APPH. Elevated levels of glyceride and cholesterol are major risk factors of potential secondary complications, such as the organ disorders associated with DM. Therefore, DF can be an effective therapeutic agent to provide protection from DM-related secondary complications. Our results also show that DF treatment effectively ameliorated the changes triggered by DM in kidneys, liver, adipose tissue, lungs, and heart, thereby effectively showing its antidiabetic potential. 

## 5. Conclusions

The results therefore indicate that DF efficiently regulates blood glucose by conserving β-cell population and retaining insulin levels and thereby protects DM mice from diabetes-associated tissue damages. Therefore, administration of DF, an active principle from APPH with antidiabetic properties, can be used as an alternative treatment strategy.
